# Surgical Management of Pediatric Foot Macrodactyly: A Case Series Highlighting Variable Growth Patterns and Soft-Tissue Challenges

**DOI:** 10.7759/cureus.117508

**Published:** 2026-10-05

**Authors:** Kosuke Shintani, Keisuke Nakagawa, Ryo Hosomi, Yuma Onishi, Chinatsu Ohira, Hidetomi Terai

**Affiliations:** 1 Department of Orthopaedic Surgery, Osaka Metropolitan University Graduate School of Medicine, Osaka, JPN; 2 Department of Pediatric Orthopaedic Surgery, Osaka City General Hospital, Osaka, JPN; 3 Department of Orthopaedic Surgery, Osaka City General Hospital, Osaka, JPN

**Keywords:** "amputation", cloves, epiphysiodesis, klippel–trenaunay syndrome, macrodactyly, osteotomy, pediatric orthopedic surgery, pik3ca-related overgrowth spectrum disorder, ray amputation, soft-tissue overgrowth

## Abstract

Macrodactyly of the foot is a rare congenital anomaly characterized by disproportionate overgrowth of osseous and soft-tissue structures. Its clinical course varies considerably among patients, and no standardized surgical strategy has been established. We retrospectively reviewed four pediatric patients with foot macrodactyly who underwent surgical treatment at our institutions.

The series included two patients without clinical features suggestive of an associated overgrowth syndrome and two with associated overgrowth syndromes. Surgical procedures were selected according to the extent and progression of the deformity and included epiphysiodesis, phalangectomy, distal toe amputation, ray resection, metatarsal shortening osteotomy, and soft-tissue debulking. One patient showed relatively limited subsequent enlargement after early surgical treatment and required no further surgery, whereas the other patients required additional procedures for progressive or residual deformity. In the two patients with associated overgrowth syndromes, marked soft-tissue hypertrophy remained a major therapeutic challenge despite surgical treatment, including soft-tissue debulking.

These cases demonstrate that surgical management of foot macrodactyly should consider not only growth behavior but also the three-dimensional characteristics of the deformity. Longitudinal and transverse enlargement can be addressed by growth modulation and skeletal procedures, whereas vertical enlargement caused predominantly by soft-tissue hypertrophy may be more difficult to control. A staged and individualized surgical strategy with long-term follow-up is therefore important, particularly in patients with progressive deformity or marked soft-tissue involvement.

## Introduction

Macrodactyly is a rare congenital anomaly characterized by disproportionate localized overgrowth of osseous and soft-tissue structures [1-3]. Although it can affect both the upper and lower extremities, macrodactyly of the foot presents distinct clinical challenges because enlargement may interfere with footwear, produce pressure-related symptoms, and ultimately impair gait and daily activities [2-4].

Somatic activating variants involving the phosphatidylinositol-3-kinase catalytic subunit alpha (PIK3CA) pathway have been identified in patients with macrodactyly [5]. Macrodactyly may occur as an isolated condition or as a manifestation of PIK3CA-related overgrowth spectrum (PROS), which includes CLOVES syndrome and other segmental overgrowth disorders [6,7]. However, molecular confirmation is not always available in individual patients, and diagnosis may therefore be based on clinical findings. Nevertheless, its clinical presentation and natural history remain highly variable.

Macrodactyly has traditionally been classified according to growth behavior into static and progressive types [1]. In the static type, the affected digit grows approximately in proportion to the remainder of the extremity, whereas in the progressive type, disproportionate enlargement continues during growth. More recent clinical series have also demonstrated differences in treatment requirements between static and progressive forms [3,4].

Various surgical procedures have been described, including epiphysiodesis, soft-tissue debulking, phalangectomy, shortening osteotomy, toe amputation, and ray resection [2-4,8-12]. However, no single procedure is appropriate for all patients, and repeated surgical interventions may be required during growth, particularly in progressive disease [3,4]. Furthermore, although skeletal enlargement can often be addressed surgically, excessive soft-tissue volume may persist even after skeletal procedures.

The purpose of this case series was to describe the clinical characteristics, surgical management, and longitudinal outcomes of four pediatric patients with foot macrodactyly and to highlight the importance of growth behavior and the three-dimensional characteristics of the deformity in surgical planning.

## Case presentation

Case 1

A seven-year-old boy presented with macrodactyly of the left foot associated with syndactyly of the second and third toes. The patient was first evaluated at our department during infancy. The overgrowth was confined to the affected foot and toes, and no clinical features suggestive of an associated overgrowth syndrome were identified. Genetic confirmation was not available.

At two years of age, he underwent syndactyly release and epiphysiodesis of the phalanges of the affected toes. Postoperatively, wound infection and flap necrosis occurred, requiring distal phalangectomy and flap reconstruction. During subsequent growth, enlargement of the affected toes was mild compared with that of the adjacent toes, and no additional surgery was required.

At the latest follow-up at seven years of age, the deformity remained relatively stable, and observation was continued. Although subsequent enlargement was relatively limited, precise classification as static or progressive was difficult because surgical intervention was performed at an early age and serial quantitative measurements of digit growth before surgery were not available (Figure 1).

**Figure 1:**
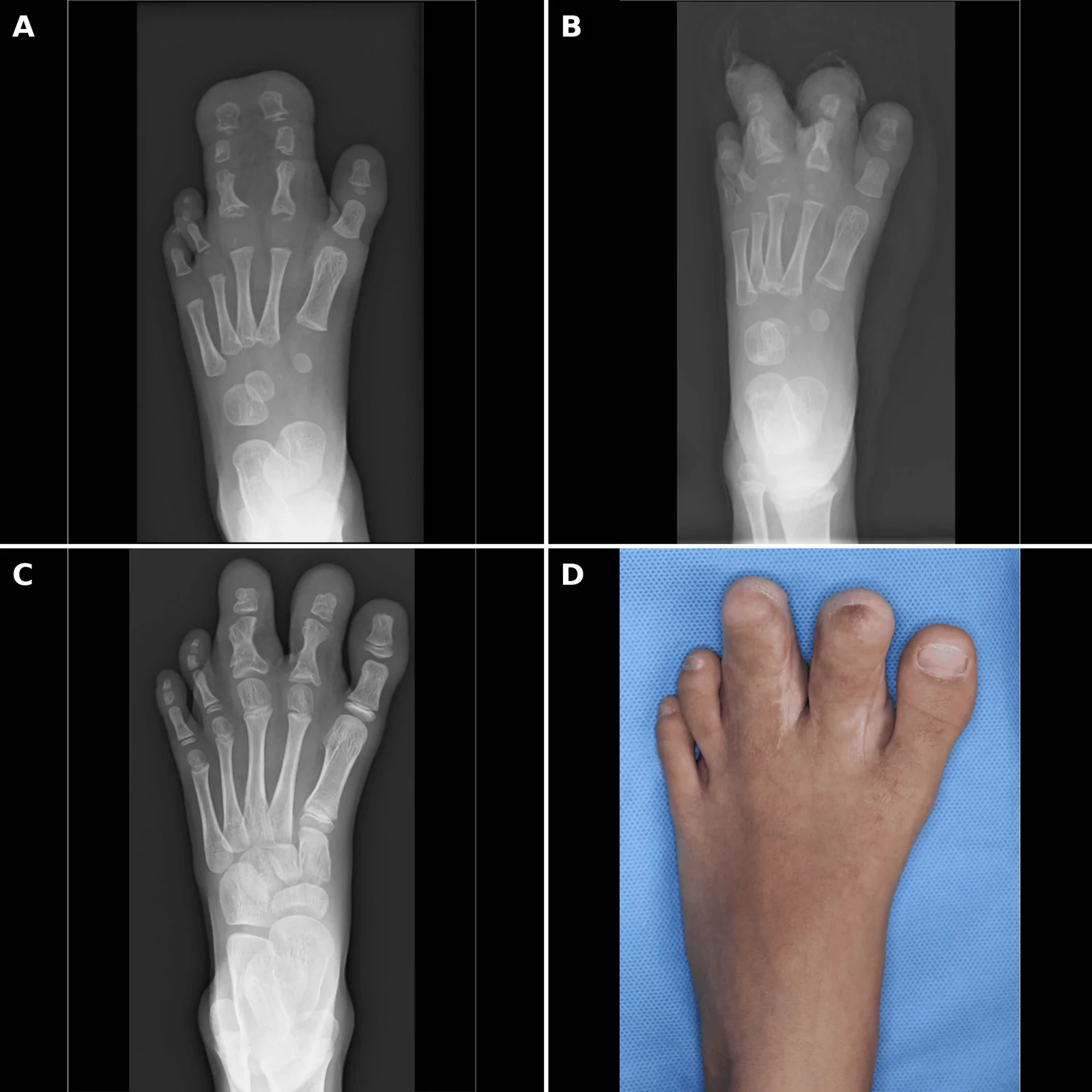
Clinical course of Case 1. (A) Initial radiograph showing macrodactyly of the second and third toes. (B) Radiograph after epiphysiodesis and syndactyly release at two years of age. Postoperative wound infection and flap necrosis subsequently required distal phalangectomy and flap reconstruction. (C) Radiograph and (D) clinical photograph at the final follow-up at seven years of age, showing relatively limited subsequent enlargement of the affected toes.

Case 2

A 15-year-old girl presented with macrodactyly of the left foot associated with syndactyly of the second and third toes. The patient was first evaluated at our department during infancy. The overgrowth was confined to the affected foot and toes, and no clinical features suggestive of an associated overgrowth syndrome were identified. Genetic confirmation was not available.

At one year of age, she underwent distal phalangectomy and syndactyly release. Because the affected toes continued to enlarge disproportionately compared with the adjacent toes, epiphysiodesis of the phalanges was performed at two years of age. Further enlargement was subsequently observed, and ray resection was performed at four years of age.

During subsequent follow-up, prominence of the residual metatarsal base became apparent. At the latest follow-up at 15 years of age, the prominence was symptomatic, and additional bone resection was planned (Figure 2).

**Figure 2:**
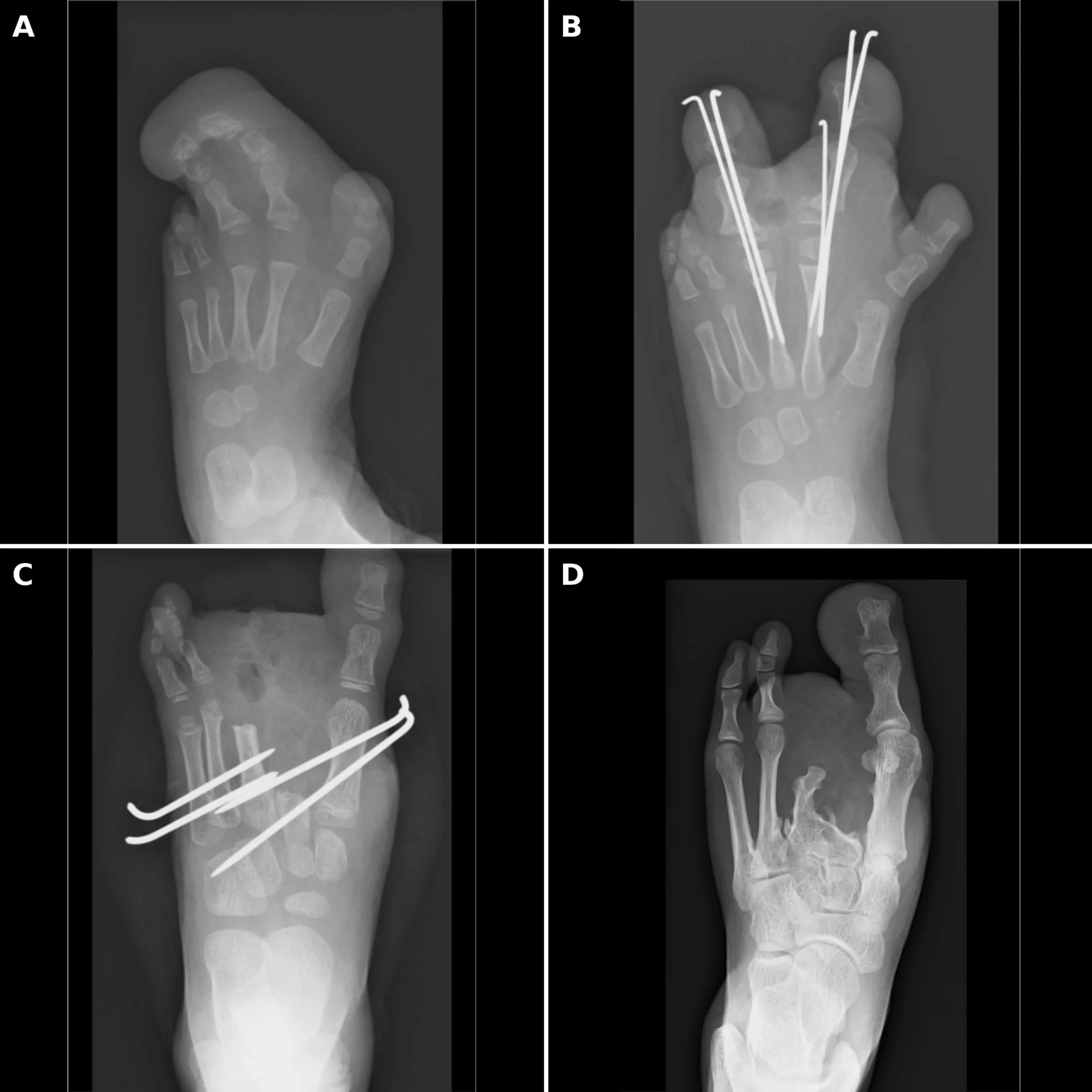
Clinical course of Case 2. (A) Radiograph at one year of age showing macrodactyly of the second and third toes. (B) Radiograph after epiphysiodesis at two years of age. (C) Radiograph after ray resection at four years of age. (D) Radiograph at 15 years of age showing prominence of the residual metatarsal base.

Although ray resection substantially reduced the enlarged digital component, this case illustrates the importance of long-term assessment of the residual skeletal configuration after reconstructive surgery.

Case 3

An eight-year-old girl with CLOVES syndrome presented with bilateral macrodactyly associated with syndactyly of the second and third toes. She was first evaluated at our department during infancy and was diagnosed with CLOVES syndrome following genetic testing. Clinically, she had marked adipose overgrowth and prominent vascular malformations, as well as a large abdominal and retroperitoneal lymphatic malformation. She underwent laser treatment for vascular malformations and received sirolimus for the extensive abdominal and retroperitoneal lymphatic malformations. She also received rivaroxaban for portal vein thrombosis and pulmonary embolism. Marked soft-tissue overgrowth was present in addition to skeletal enlargement.

Because of severe enlargement, surgical reduction was performed on the left foot at five years of age and on the right foot at six years of age. Distal portions of the enlarged toes were resected to reduce the overall size of the feet and facilitate shoe wear. Additional resection of prominent residual portions of the great and fifth toes was subsequently required.

Despite these procedures, marked soft-tissue hypertrophy persisted. Further soft-tissue debulking was considered; however, the prominent vascular malformations were associated with substantial intraoperative bleeding, making extensive soft-tissue resection technically challenging.

At the latest follow-up at eight years of age, excessive soft-tissue thickness remained the principal residual deformity (Figure 3).

**Figure 3:**
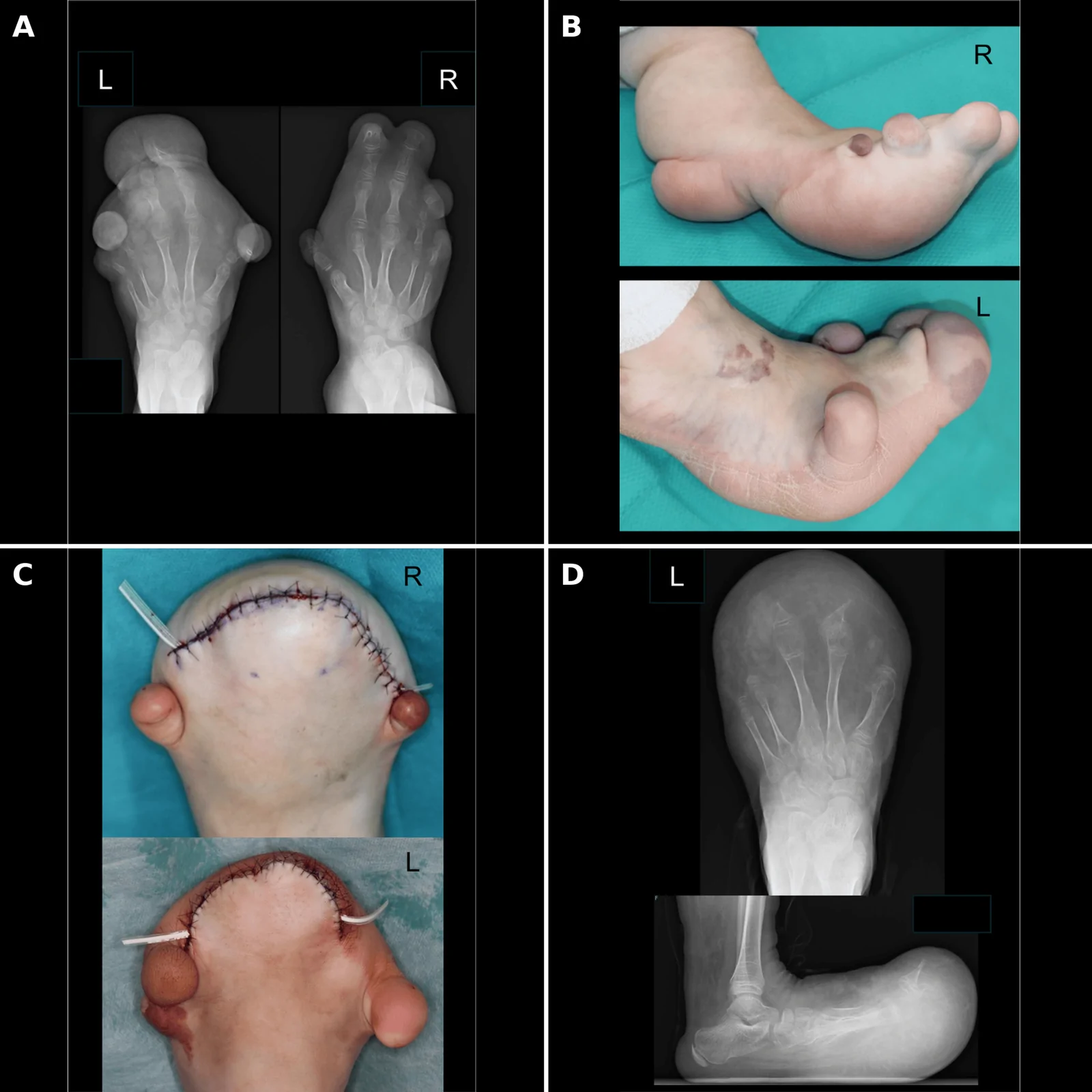
Clinical course of Case 3 with CLOVES syndrome. (A) Preoperative radiographs showing severe bilateral macrodactyly. (B) Preoperative clinical photographs demonstrating marked soft-tissue overgrowth of both feet. (C) Postoperative clinical photographs after distal resection of the enlarged toes. (D) Anteroposterior and lateral radiographs of the left foot at eight years of age, demonstrating persistent soft-tissue hypertrophy and substantial vertical thickness.

Case 4

An 11-year-old boy with Klippel-Trenaunay syndrome presented with bilateral macrodactyly associated with syndactyly of the second and third toes. The patient was first evaluated at our department at two years of age. The diagnosis was based on bilateral lower-limb overgrowth, more pronounced on the left side, and superficial venous abnormalities identified by ultrasonography, without genetic confirmation. The patient also had left gluteal overgrowth, for which soft-tissue debulking was performed by plastic surgeons. No other apparent comorbidities were identified. Marked soft-tissue overgrowth was present in addition to skeletal enlargement.

He underwent syndactyly release of the right foot at one year of age at another institution. At four years of age, third-ray resection, epiphysiodesis of the affected phalanges and metatarsals, and extensive soft-tissue debulking were performed because of progressive enlargement. Despite substantial reduction in foot size, disproportionate enlargement recurred during subsequent growth.

At 10 years of age, additional surgery consisting of distal toe amputation, metatarsal shortening osteotomy, and extensive soft-tissue debulking was performed to reduce the overall size of the affected foot. Despite substantial soft-tissue reduction at surgery, soft-tissue overgrowth remained a concern during subsequent follow-up.

At the latest follow-up at 11 years of age, further soft-tissue debulking was being considered because of persistent soft-tissue enlargement (Figure 4). Although walking ability was not impaired, the marked enlargement of the feet made it difficult to wear commercially available footwear, and custom-made footwear was required.

**Figure 4:**
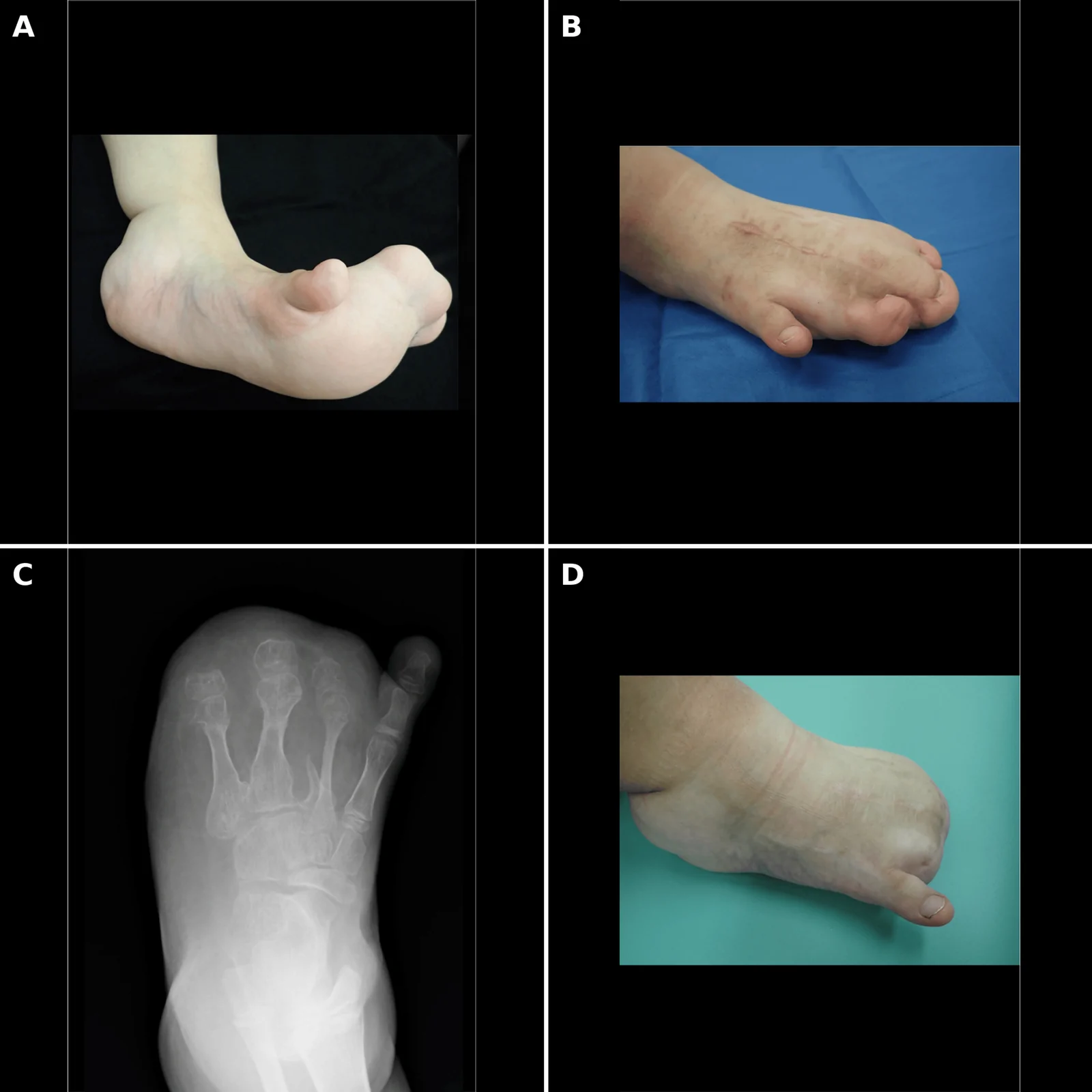
Clinical course of Case 4 with Klippel-Trenaunay syndrome. (A) Clinical appearance at four years of age before third-ray resection, epiphysiodesis, and extensive soft-tissue debulking. (B) Clinical appearance after these procedures at four years of age, showing substantial reduction in foot size. (C) Radiograph and (D) clinical photograph at the latest follow-up at 11 years of age, after additional distal toe amputation, metatarsal shortening osteotomy, and extensive soft-tissue debulking at 10 years of age, showing recurrent soft-tissue overgrowth.

## Discussion

Macrodactyly of the foot presents a difficult reconstructive problem because of its rarity, variable growth behavior, and heterogeneous involvement of osseous and soft-tissue structures [2-4,8-12]. The principal goals of treatment are to achieve a foot of acceptable size and appearance, permit comfortable shoe wear, and prevent pain, callosity, and gait disturbance [2-4,8-10,12]. In the present series, the clinical courses differed considerably among the four patients, emphasizing the importance of individualized treatment planning.

One important factor in surgical decision-making is the growth pattern. Barsky classified macrodactyly into static and progressive types [1]. In the static type, the affected structures grow at approximately the same rate as the unaffected extremity, whereas the progressive type demonstrates disproportionate enlargement over time. In a recent series of 24 feet in 19 children, Rougereau et al. reported that progressive forms required significantly more surgical procedures than static forms [4]. In our series, Case 1 showed relatively limited enlargement following early surgical treatment and required no additional surgery during the observation period. The other cases required additional procedures for progressive or residual deformity. However, strict categorization into static and progressive types may be difficult, particularly because surgical intervention itself alters the subsequent growth of the affected structures. Therefore, serial assessment of actual growth behavior remains important when planning treatment.

A second important consideration is the three-dimensional nature of the deformity. For surgical planning, we considered enlargement in longitudinal, transverse, and vertical dimensions. Longitudinal overgrowth may be addressed by epiphysiodesis, phalangectomy, distal toe amputation, or shortening osteotomy, whereas excessive transverse width may be reduced by ray resection [2-4,8-12]. Ray resection and toe amputation have been reported to achieve substantial reductions in foot size with satisfactory functional outcomes in appropriately selected patients [8-10]. These procedures can therefore reduce the skeletal dimensions of the foot and facilitate shoe wear.

Vertical enlargement, however, represents a different challenge because it is often predominantly related to soft-tissue hypertrophy. This was particularly evident in Cases 3 and 4. Despite substantial reduction of the skeletal components, excessive soft-tissue thickness remained and continued to affect the overall size and shape of the foot. Thus, correction of longitudinal and transverse skeletal enlargement does not necessarily result in adequate reduction of the vertical dimension or total foot volume.

The distribution of osseous and soft-tissue overgrowth also differed among our patients. In Cases 1 and 2, the overgrowth was confined to the affected foot and toes, and no clinical features suggestive of an associated overgrowth syndrome were identified. In contrast, Cases 3 and 4 had associated overgrowth syndromes and demonstrated marked soft-tissue hypertrophy in addition to skeletal enlargement. Although the small number of patients does not allow conclusions regarding differences between syndromic and non-syndromic macrodactyly, these observations illustrate the importance of assessing the relative contributions of skeletal and soft-tissue components in each patient. Large clinical series have similarly demonstrated considerable heterogeneity in the tissues and anatomical regions involved in foot macrodactyly [8].

Management of marked soft-tissue hypertrophy may be particularly challenging when vascular abnormalities coexist. In Case 3, CLOVES syndrome was associated with vascular malformations, limiting the extent of safe soft-tissue debulking because of the potential for substantial intraoperative bleeding. CLOVES syndrome is caused by somatic mosaic activating variants in PIK3CA and is characterized by complex segmental overgrowth that may include adipose and vascular abnormalities [6,7]. Extensive reduction of soft-tissue volume must therefore be balanced against the risks of vascular injury, bleeding, wound complications, and compromised tissue viability.

In Case 3, sirolimus was administered for extensive vascular and lymphatic malformations associated with CLOVES syndrome. Despite its potential to suppress soft-tissue overgrowth, substantial foot enlargement persisted and required surgical treatment. In Case 4, no pharmacologic or molecularly targeted therapy was administered for the foot overgrowth. In both cases, although walking ability was not impaired, custom-made footwear was required because commercially available shoes could not accommodate the enlarged feet. Repeated surgical debulking was therefore performed to reduce foot volume and improve footwear accommodation.

The timing and extent of skeletal procedures also require careful consideration. Epiphysiodesis performed during childhood may help control longitudinal growth when sufficient growth potential remains, but subsequent procedures may still be required if disproportionate enlargement continues [4,11]. Ray resection can substantially reduce the transverse dimension of the foot [8-10], but long-term follow-up is necessary to assess the residual skeletal configuration as the child grows. In Case 2, prominence of the residual metatarsal base became symptomatic during long-term follow-up and additional bone resection was planned. The present case series does not allow us to determine whether this prominence resulted primarily from subsequent growth, the configuration remaining after the initial ray resection, or a combination of these factors.

Taken together, our cases suggest that surgical planning for pediatric foot macrodactyly should integrate growth behavior and the three-dimensional distribution of the deformity. Longitudinal and transverse skeletal enlargement may be addressed through growth modulation and skeletal reduction, whereas vertical enlargement related to soft-tissue hypertrophy may remain difficult to control. Associated overgrowth syndromes and vascular abnormalities should also be considered because they may influence both the characteristics of the deformity and the feasibility of soft-tissue reduction. Rather than relying on a single definitive procedure, treatment should therefore be individualized, with staged procedures and, when necessary, extensive soft-tissue debulking considered according to the severity and distribution of the overgrowth [2,4,9-11].

This study has several limitations. First, it included only four patients because of the rarity of the condition. Second, the patients differed in age, associated conditions, severity, and surgical procedures, preventing direct comparison of treatment outcomes. Third, quantitative functional and patient-reported outcome measures were not available. Standardized serial measurements of foot or toe dimensions were not routinely recorded, and the available clinical photographs and radiographs were obtained under nonuniform conditions; therefore, reliable retrospective quantitative comparison of longitudinal, transverse, and vertical dimensions could not be performed. In Cases 1 and 2, the original evaluations were not performed using a standardized syndromic assessment protocol, and genetic confirmation was not available; therefore, associated overgrowth syndromes could not be definitively excluded. Detailed molecular data regarding the genetic diagnosis of CLOVES syndrome in Case 3, including the gene and variant, sample used, testing method, variant classification criteria, and exact age at diagnosis, were not available for retrospective review. Finally, classification of growth behavior was based primarily on serial clinical and radiographic observations rather than standardized longitudinal measurements. Nevertheless, the different long-term clinical courses of these four patients illustrate practical considerations relevant to surgical planning for pediatric foot macrodactyly.

## Conclusions

Pediatric foot macrodactyly demonstrates considerable variability in growth behavior and in the relative contributions of osseous and soft-tissue overgrowth. Surgical treatment may therefore need to be individualized according to the progression and three-dimensional characteristics of the deformity.

Longitudinal and transverse enlargement can often be addressed with growth modulation and skeletal procedures such as epiphysiodesis, phalangectomy, distal toe amputation, ray resection, and shortening osteotomy. In contrast, vertical enlargement caused predominantly by soft-tissue hypertrophy remains particularly difficult to manage and may require extensive soft-tissue debulking, especially in patients with associated overgrowth syndromes. When vascular abnormalities are present, however, the extent of soft-tissue resection must be carefully balanced against the risk of bleeding and other complications. Careful long-term follow-up is important because residual or progressive deformity may require additional treatment as the child grows. These cases illustrate that a staged surgical strategy may be considered when necessary rather than expecting a single procedure to provide definitive correction.

## References

[1] Barsky AJ (1967). Macrodactyly. J Bone Joint Surg Am.

[2] Chang CH, Kumar SJ, Riddle EC, Glutting J (2002). Macrodactyly of the foot. J Bone Joint Surg Am.

[3] Chen W, Tian X, Chen L, Huang W (2021). Clinical characteristics of 93 cases of isolated macrodactyly of the foot in children. J Orthop Surg Res.

[4] Rougereau G, Fitoussi F, Marty-Diloy T, Bachy M, Vialle R, Langlais T (2023). Strategy and clinical outcomes of child foot surgery for macrodactyly. Orthop Traumatol Surg Res.

[5] Rios JJ, Paria N, Burns DK, Israel BA, Cornelia R, Wise CA, Ezaki M (2013). Somatic gain-of-function mutations in PIK3CA in patients with macrodactyly. Hum Mol Genet.

[6] Kurek KC, Luks VL, Ayturk UM (2012). Somatic mosaic activating mutations in PIK3CA cause CLOVES syndrome. Am J Hum Genet.

[7] Keppler-Noreuil KM, Rios JJ, Parker VE (2015). PIK3CA-related overgrowth spectrum (PROS): diagnostic and testing eligibility criteria, differential diagnosis, and evaluation. Am J Med Genet A.

[8] Bulut M, Karakurt L, Belhan O, Serbest S (2011). Ray amputation for the treatment of macrodactyly in the foot: report of three cases. Acta Orthop Traumatol Turc.

[9] Hop MJ, van der Biezen JJ (2011). Ray reduction of the foot in the treatment of macrodactyly and review of the literature. J Foot Ankle Surg.

[10] Kim J, Park JW, Hong SW, Jeong JY, Gong HS, Baek GH (2015). Ray amputation for the treatment of foot macrodactyly in children. Bone Joint J.

[11] Gluck JS, Ezaki M (2015). Surgical treatment of macrodactyly. J Hand Surg Am.

[12] Hart ES, Grottkau BE (2012). Isolated macrodactyly of the foot: diagnosis and treatment. Orthop Nurs.

